# Synergetic Photocatalytic Peroxymonosulfate Oxidation of Benzotriazole by Copper Ferrite Spinel: Factors and Mechanism Analysis

**DOI:** 10.3390/toxics11050429

**Published:** 2023-05-04

**Authors:** Masoumeh Golshan, Na Tian, Gcina Mamba, Babak Kakavandi

**Affiliations:** 1 Department of Environmental Health Engineering, Faculty of Health, Zabol University of Medical Sciences, Zabol 9861615881, Iran; 2 School of Environmental Studies, China University of Geosciences, Wuhan 430074, China; 3 Unidad Docente Ingeniería Sanitaria, Departamento de Ingeniería Civil: Hidráulica, Energía y Medio Ambiente, E.T.S. de Ingenieros de Caminos, Canales y Puertos, Universidad Politécnica de Madrid, s/n, 28040 Madrid, Spain; 4 Institute for Nanotechnology and Water Sustainability, College of Science, Engineering and Technology, University of South Africa, Johannesburg 1709, Florida, South Africa; 5 Research Center for Health, Safety and Environment, Alborz University of Medical Sciences, Karaj 3149779453, Iran; 6 Department of Environmental Health Engineering, Alborz University of Medical Sciences, Karaj 3149779453, Iran

**Keywords:** PMS activation, copper ferrite, photocatalyst, BTA

## Abstract

The development of oxidation processes with the efficient generation of powerful radicals is the most interesting and thought-provoking dimension of peroxymonosulfate (PMS) activation. This study reports the successful preparation of a magnetic spinel of CuFe_2_O_4_ using a facile, non-toxic, and cost-efficient co-precipitation method. The prepared material exhibited a synergetic effect with photocatalytic PMS oxidation, which was effective in degrading the recalcitrant benzotriazole (BTA). Moreover, central composite design (CCD) analysis confirmed that the highest BTA degradation rate reached 81.4% after 70 min of irradiation time under the optimum operating conditions of CuFe_2_O_4_ = 0.4 g L^−1^, PMS = 2 mM, and BTA = 20 mg L^−1^. Furthermore, the active species capture experiments conducted in this study revealed the influence of various species, including ^•^OH, SO_4_^•−^, O_2_^•−,^ and *h*^+^ in the CuFe_2_O_4_/UV/PMS system. The results showed that SO_4_^•−^ played a predominant role in BTA photodegradation. The combination of photocatalysis and PMS activation enhanced the consumption of metal ions in the redox cycle reactions, thus minimizing metal ion leaching. Additionally, this maintained the reusability of the catalyst with reasonable mineralization efficiency, which reached more than 40% total organic carbon removal after four batch experiments. The presence of common inorganic anions was found to have a retardant effect on BTA oxidation, with the order of retardation following: HCO_3_^−^ > Cl^−^ > NO_3_^−^ > SO_4_^2−^. Overall, this work demonstrated a simple and environmentally benign strategy to exploit the synergy between the photocatalytic activity of CuFe_2_O_4_ and PMS activation for the treatment of wastewater contaminated with widely used industrial chemicals such as BTA.

## 1. Introduction

Benzotriazole (BTA) is an emerging contaminant originating from a wide range of industrial and domestic applications, such as the protection of alloys against corrosion, automotive cooling and aircraft de-icing fluids, enhancement of fastness to light in fabrics, antifreezes, and the production of various detergents. BTA is a cyclic compound that has a benzene ring that is fused into a C-C bond with three nitrogen atoms [1,2]. The presence of BTA has been reported in effluent-receiving water bodies (~7–18 μg/L) [3], seawater, and urban runoff as the major sources in the natural environment [4]. Generally, BTA has a low sorption affinity on organic matter, is extremely soluble in water, and is polar, which is responsible for its high environmental mobility [5]. Consequently, its persistence and toxicity in the aquatic environment make it less susceptible to chemical-biological treatment processes [4,5,6]. Moreover, TBA has been reported as a potential human carcinogen and is toxic to aquatic organisms. Therefore, there is an urgent need to explore efficient water treatment technologies for BTA mineralization.

Advanced oxidation processes (AOPs) have enormous potential for eliminating emerging and refractory pollutants using various reactive oxygen species (ROS) such as singlet oxygen (^1^O_2_) molecules, sulfate (SO_4_^•−^), hydroxyl (^•^OH), and superoxide (O_2_^•−^) radicals. Several AOPs, including Fenton- and photo-Fenton-like systems [7], photocatalytic reactions [2], ozonation, and ultrasound-assisted oxidation [8] have been used for oxidative BTA elimination. Sulfate based-AOPs such as peroxymonosulfate (HSO_5_^−^, PMS) and peroxydisulfate (S_2_O_8_^2−^, PDS)-mediated oxidation have emerged as promising and advantageous AOPs, owing to their flexible operational conditions. For example, sulfate-based AOPs are applicable over a wide pH range, unlike the Fenton process, which is effective over a narrow pH range in the acidic region [9]. PMS is considered to be environmentally benign because of its less harmful by-products (SO_4_^2−^) [10]. Furthermore, SO_4_^•−^ has higher oxidizing efficiency (*E*^0^ = 2.5–3.1 V) vs. ^•^OH (*E*^0^ = 1.9–2.7 V) at neutral pH, better selectivity, and higher persistency in aqueous solutions (30–40 µs), allowing longer contact times with recalcitrant pollutants. Additionally, SO_4_^•−^ is less prone to interfering with water constituents, which is ideal for water and wastewater treatment [11]. Therefore, SO_4_^•−^ can overcome the unescapable deficiencies of conventional AOPs [12]. In particular, the activation of the PMS or PDS precursors by UV or ultrasound irradiation, base and transition metal catalysts ( such as Fe^2+^, Mn^2+^, Co^2+^, Cu^2+^, etc.), heat, and carbon-based materials can produce SO_4_^•−^ via electron transfer, as shown in Equation (1) [13,14]. In this regard, homogeneous transition-metal-catalyzed PMS activation is often preferred due to its ease of initiation at room temperature and pressure, requiring low energy as shown in Equations (2) and (3) to form SO_4_^•−^ as the predominant oxidizing species [15]:

S_2_O_8_^2−^/HSO_5_^−^ → SO_4_^•−^ + (HSO_5_^•^, SO_4_^2−^, ^•^OH) (1)

HSO_5_^−^ + e^−^ → SO_4_^•−^ + OH^−^ (2)

HSO_5_^−^ + e^−^ → SO_4_^2−^ + ^•^OH (3)

However, the homogeneous transition metal ion-mediated PMS activation system results in secondary pollution due to the difficulty of recovering the potentially toxic transition metal ions [16]. Contrary to this, heterogeneous catalytic PMS activation offers several advantages, such as recoverability and structural stability, low toxicity of catalysts, and minimal secondary pollution [17]. Given the difficulty associated with recovering nanocatalysts, magnetic Fe-based catalysts have been investigated due to their easy separation under an external magnetic field [18,19]. The heterogeneous catalytic activation of PMS largely depends on the interaction between PMS and the catalyst. As a result, a large specific surface area, excellent catalytic activity, and a highly porous structure are needed so that the activation rate of PMS can be improved [20]. Copper ferrites (CuFe_2_O_4_), which possess intrinsic surface hydroxyl sites and ferromagnetic properties, have been extensively studied for their effective role in activating PMS for the removal of refractory organic pollutants in water [21,22]. Despite its relatively low catalytic effectiveness compared to CoFe_2_O_4_, the low toxicity of CuFe_2_O_4_ makes it an important PMS activator. Furthermore, a significant linear correlation between the degradation rate and quantity of surface hydroxyl sites has been demonstrated [23]. More specifically, CuF_2_O_4_ as a Cu–Fe mixed-metal catalyst endows the surface with synergistic redox reactions for Cu^3+^/Cu^2+^, Cu^+^/Cu^2+^, and Fe^2+^/Fe^3+^ in a tetrahedral and/or octahedral structure to effectively generate SO_4_^•−^ radicals from PMS. The photocatalytic PMS activation by CuFe_2_O_4_ stems from its suitable light absorption, which provides the photosynergistic effect that is favorable for organic pollutants' degradation [22,24]. In this context, the photogenerated electrons are donated to the PMS electron acceptor (electron transfer), which cleaves the O-O bond in the PMS molecule into SO_4_^•−^ and ^•^OH radicals [25]. Furthermore, the recombination phenomenon of photoinduced charges is suppressed through the consumption of photoinduced electrons by PMS, leading to an amplification of the catalytic efficiency [13,26]. In addition, ion leaching in aqueous environments is particularly minimized due to the strong interaction of bimetals and the convenient recovery of the catalyst after its application.

Therefore, this study leveraged the intrinsic properties of CuFe_2_O_4_, including its ability to absorb UV photons and catalyze PMS activation, to construct a CuFe_2_O_4_/UV/PMS reaction system. Such an integrated process of CuFe_2_O_4_/UV/PMS without sludge generation and secondary pollution is attractive for the fast and efficient removal of BTA from water. Several studies applied the CuFe_2_O_4_/PMS process and reported promising results on the catalytic oxidation of different micropollutants at neutral pH [27,28,29,30]. However, the exploitation of the well-integrated CuFe_2_O_4_/UV/PMS for the removal of BTA has been rarely reported. Therefore, we used a simple and environmentally friendly co-precipitation method to obtain CuFe_2_O_4_ nanoparticles and used it to activate PMS for BTA degradation under UV irradiation. The structural, morphological, and physicochemical properties of CuFe_2_O_4_ were investigated. We first studied the activity of the different reaction systems toward BTA degradation and found that the UV-assisted CuFe_2_O_4_/PMS combination transcended other combinations. Then, the interactive effect of operational parameters, including PMS dosage, catalyst dosage, different BTA concentrations, and reaction time, on the catalytic performance was optimized using response surface methodology (RSM) via central composite design (CCD). The reusability and stability of CuFe_2_O_4_ were consecutively analyzed through four cycles, and the treatment of BTA in the presence of interfering water constituents was evaluated under the optimum conditions. Finally, the probable transformation pathways of BTA were explored. This study provides a simple strategy for coupling UV and catalytic PMS activation over a magnetically separable catalyst to ensure high catalytic performance for the elimination of organic pollutants and easy recovery of the catalyst.

## 2. Experimental Section

### 2.1. Chemicals

Benzotriazole (C_6_H_5_N_3_, 99%), copper nitrate trihydrate (Cu(NO_3_)_2_·3H_2_O, 99%), and ferric nitrate nonahydrate (Fe(NO_3_)_3_·9H_2_O, 99%) were purchased from Merck Inc. Potassium peroxymonosulfate (Oxone, KHSO_5_·0.5KHSO_4_·0.5K_2_SO_4_), *tert*-butyl alcohol (TBA, (CH_3_)_3_COH), *p*-Benzoquinone (BQ, C_6_H_4_O_2_), methanol (MeOH, CH_3_OH), and potassium iodide (KI) were obtained from Sigma-Aldrich Inc., (St. Louis, MO, USA). All chemicals were used as received without further purification, and deionized water (DI-water) was used for all aqueous solutions.

### 2.2. Preparation of Catalyst

Magnetic CuFe_2_O_4_ nanoparticles were prepared using a co-precipitation method from the precursors Cu(NO_3_)_2_·3H_2_O and Fe(NO_3_)_3_·9H_2_O in a 1:2 molar ratio as previously reported [31].

### 2.3. Material Characterization

Powder X-ray diffraction (XRD) was performed on a Quantachrome/NOVA 2000X-ray diffractometer with Cu *Kα* radiation (λ = 1.54 nm), operated at 30 mA and 40 kV in a 2θ range of 10° to 80°. Brunauer-Emmett-Teller analysis (BET, ASAP 2010; Micromeritics, Norcross, GA, USA) was applied to determine the surface area, average pore size, and volume. The magnetic features of the catalyst were studied using a vibrating sample magnetometer (VSM, Lakeshore 7400, Westerville, OH, USA). Meanwhile, the detailed microstructure of the catalyst was investigated using transmission electron microscopy (TEM, JEM-2100; Jeol; Akishima, Tokyo, Japan) with high resolution at 100 kV. A field emission scanning electron microscope (FESEM, Mira 3-XMU, Tescan USA Inc., Warrendale, PA, USA) coupled with an energy dispersive X-ray spectrometer (EDS) device was exploited for the morphology and qualitative chemical analysis of the catalyst. Optical response measurements were conducted using a UV–Vis diffuse reflectance spectrometer (DRS, Hitachi Ltd., Chiyoda, Tokyo, Japan) in the wavelength range of 190 to 800 nm.

### 2.4. Degradation Experiments and Sample Analyses

A cylindrical 300 mL quartz photoreactor equipped with a 6.0 W Hg UV-C lamp (Philips, The Netherlands) with a luminous intensity of 7800 cd and fitted with a 254 nm filter was used for the photocatalytic/PMS experiments at 25 ± 5 °C. The initial pH in all experiments (unless stated otherwise) was that of the BTA solution without adjustment. For each experiment, a certain mass of catalyst was put into the BPA solution (200 mL) and mechanically stirred (RW 20, IKA, Staufen im Breisgau, Germany) at 250 r/min for 60 min to ensure the adsorption-desorption equilibrium of the BTA molecules on the catalyst surface. Thereafter, a specific amount of PMS was added, and the light source was switched on to initiate the photocatalytic/PMS BTA oxidation reaction. At regular intervals, 2 mL of BTA solution was withdrawn, the catalyst was magnetically separated, and 1 mL of Na_2_S_2_O_3_. 5H_2_O (20 mM) was added before analysis using high-performance liquid chromatography (HPLC, KNUER, Berlin, Germany). The HPLC was equipped with a C18 separation column (100–5; 4.6 mm × 250 mm, 5 µm), a 2500 UV–visible detector, and BTA measurements were conducted at a maximum wavelength of 254 nm. The mobile phase was a 50:50% (*v*/*v*) water-acetonitrile mixture with a flow rate of 1.0 mL min^−1^. The degradation efficiency (%) was used to describe the performance of the process according to Equation (4):

(4) $$Degradation\;efficiency\;\left(\%\right)=\frac{\left(C_{0}-C_{t}\right)}{C_{0}}\times 100(\%)$$

where *C*_0_ and *C_t_* are the BTA concentrations at the initial and certain treatment times, respectively. The photochemical stability of the catalyst was assessed in four repeated experiments. An atomic absorption spectrophotometer (AAS) (Analytikjena vario 6, Jena, Thueringen, Germany) was used to determine the concentrations of Fe and Cu in the filtrate for each cycle. Additionally, for each cycle, total organic carbon (TOC) determination was conducted to monitor the mineralization efficiency using a Shimadzu V_CHS/CSN_, Japan. Potassium iodide (KI), benzoquinone (BQ), *tert*-butyl alcohol (TBA), and methanol (MeOH) were applied as quenchers of the ROS. The effect of various anions existing in natural water matrices was studied during BTA removal.

### 2.5. Response Surface Method Experimental Design and Data Analysis

To determine the effect of the main factors on optimal degradation conditions of BTA, central composite design (CCD) was used under response surface methodology (RSM). In this experiment, the catalyst loading (*x*_1_), PMS dosage (*x*_2_), BTA concentration (*x*_3_), and irradiation time (*x*_4_) were varied at five various coded levels, as indicated in Table 1. The coded value of the *i*th variable (*x_i_*) was described by Equation (5):

(5) $$x_{i}=\frac{X_{i}-X_{0}}{{∆X}_{i}}$$

where *X_i_* is the actual value of the variables; *X*_0_ is *X_i_* at the center point; and ∆*X_i_* is the step with the maximum and minimum values of *X_i_*. With the application of the CCD matrix, 30 tests (2^4^ = 16 factorial points, 2 × 4 = 8 axial points, and 6 replications at the center points) were determined for four input variables (Table S1). The quadratic polynomial Equation 6 can be used to estimate the effects of four independent variables on the efficiency of the photocatalytic PMS activation process. Here, *y* is a predicted response variable of BTA degradation efficiency, *b*_0_ is the intercept, *b_i_*, *b_ij_*, and *b_ii_* are the linear, interaction, and quadratic regression coefficients, respectively, and ε is residual error.

(6) $${y\left(BTA\%\right)}_{predicted}=b_{0}+\sum b_{i}x_{i}+\sum b_{ij}x_{i}x_{j}+\sum b_{ii}{x_{i}}^{2}+\varepsilon$$

The significance and adequacy of the quadratic regression model were estimated by analysis of variance (ANOVA). The R-squared (R^2^) and the adjusted R^2^ (R^2^_adj_) values were the criteria for verifying the statistical significance of the second-order model.

## 3. Results and Discussion

### 3.1. CuFe_2_O_4_ Spinel Morphology and Microstructure Analysis

**Figure 1:**
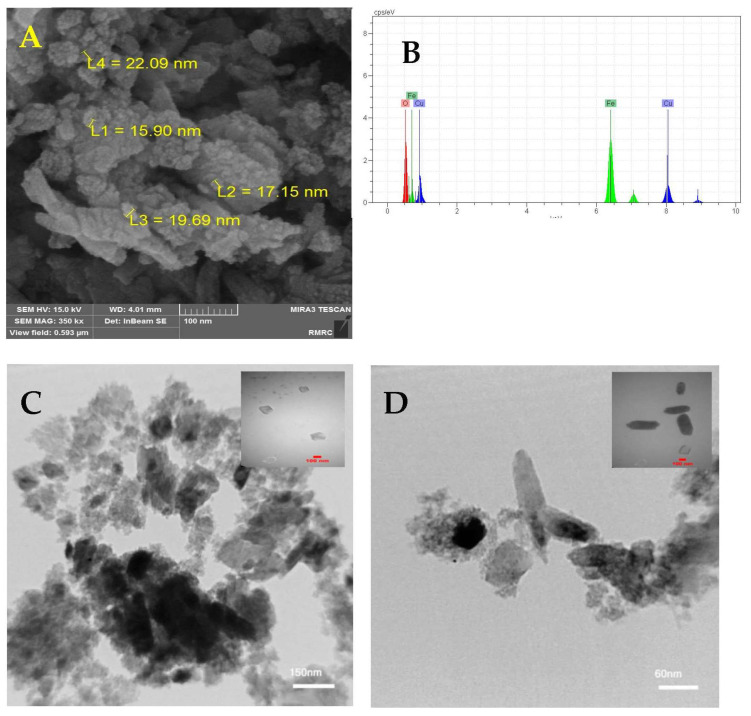
FESEM image (**A**) and EDS spectrum (**B**) of CuFe_2_O_4_; TEM micrographs of CuFe_2_O_4_ (**C**,**D**) with different scale bars.

To investigate the microstructure and morphology of pure CuFe_2_O_4_, FESEM, and TEM techniques were conducted. Figure 1A shows a FESEM image of the catalyst, which confirms an irregular polygon morphology and a slightly agglomerated state emanating from the nanoscale size of the CuFe_2_O_4_ (<26 nm). Obviously, an uneven and rough surface was witnessed from the TEM image of the CuFe_2_O_4_ catalyst (Figure 1C,D), which is favorable for the diffusion of reactants (organic pollutant and oxidant) and increase interaction with active sites of the catalyst. Ultimately, this accelerates the redox reaction on the catalyst surface, leading to pollutant degradation. In addition, EDS analysis (Figure 1B) shows that the catalyst contained the elements Cu (25.98%), Fe (47.41%), and O (26.62%) in a molar ratio of nearly 1:1.82, which was almost equal to the molar ratio of the iron and copper precursors used in the preparation of the CuFe_2_O_4_ catalyst.

### 3.2. Textural and Surface Area, Magnetic and Optical Properties

**Figure 2:**
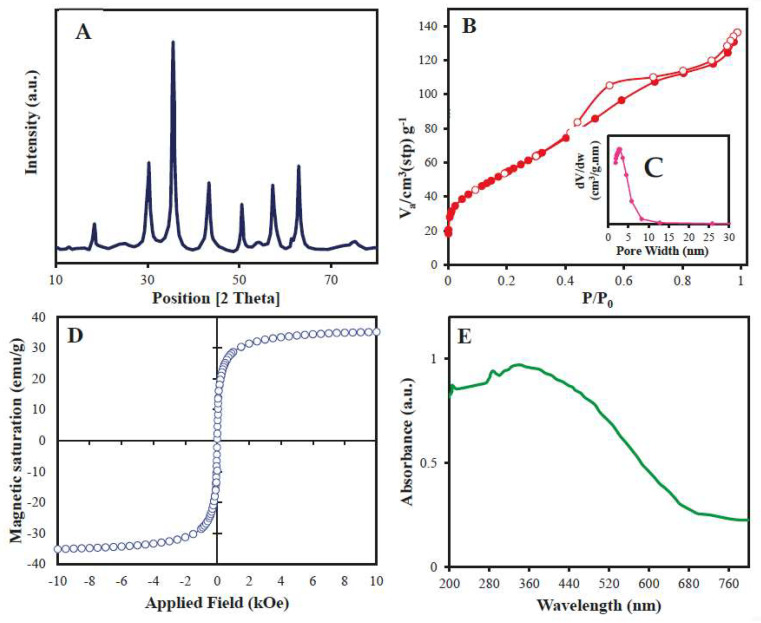
(**A**) XRD patterns, (**B**) N_2_ adsorption-desorption isotherms (insert: pore size distribution), (**C**,**D**) magnetic hysteresis loops, and (**E**) UV−Vis absorption spectra of CuFe_2_O_4_. In (**B**), it is possible to include the reference diffractogram for CuFe_2_O_4_ for easy comparison.

The crystalline phase composition of CuFe_2_O_4_ was determined by XRD (Figure 2A). The main peaks observed at 2θ of 18.22°, 30.30°, 35.85°, 43.32°, 57.39°, and 62.87° were assigned to crystal planes of (1 1 1), (2 2 0), (3 1 1), (4 0 0), (5 1 1), and (4 4 0), which are characteristic of the spinel ferrite structure of CuFe_2_O_4_ (JCPDS no. 25-0283) [21]. As demonstrated by XRD patterns, the narrow and strong diffraction peaks are indicative of the high crystallinity of the catalyst, which could be expected to exhibit high activity [27]. Furthermore, the absence of additional XRD peaks is an indication of the phase purity of the prepared material. Accordingly, the Debye–Scherrer equation was used to calculate the crystalline size of CuFe_2_O_4_ nanoparticles. The equation is given as *D = Kλ/βcosθ*, where K is the Scherrer constant (value 0.94), *λ* is the X-ray wavelength (0.154 nm), *β* is the FWHM (full width at half maximum) of photocatalysts, D is the calculated crystalline size, and θ is the diffraction angle. Using this equation, the average crystallite size of CuFe_2_O_4_ nanoparticles was calculated at 16.7 nm.

The N_2_ adsorption–desorption isotherm plot (Figure 2B) and the pore size distribution (Figure 2C) were measured at −196.88 °C. The Langmuir isotherm is related to a type IV curve in the IUPAC classification with a hysteresis loop at the relative pressure (P/P_0_) range between 0.4 and 1. This confirms that the catalyst has a mesoporous structure. Accordingly, the BET surface area of the pure CuFe_2_O_4_ nanoparticles (insert in Figure 2B) was calculated to be 201.898 m^2^ g^−1^, which indicates a considerable surface area and significant adsorption capacity for the as-prepared catalyst. The total pore volume and micropore volume were 0.191 and 0.0126 mL g^−1^, respectively, and the average pore diameter was 3.7 nm. The pore size distribution in the range of 1–30 nm manifests a mesoporous structure with an average size of about 2.6 nm (Figure 2C). Therefore, the large BET surface area with many micropores and mesopores could provide a rich source of surface reaction sites to activate PMS and also adsorb BTA [32].

The VSM technique was applied in the presence of a magnetic field ranging from −40 to +40 kOe to study the magnetic properties of CuFe_2_O_4_ (Figure 2D). The saturation magnetization value (Ms) of CuFe_2_O_4_ was found to be 35.2 emu g^−1^. Therefore, it is expected that CuFe_2_O_4_ as an ideal ferromagnetism can be easily separated from the aqueous matrix within a short time using an external magnetic field. This ensures high-quality recycling and reuse of the nanocatalyst with minimum risks of secondary contamination of the environment and simplifies the practical application of these nanoparticles. The light absorption capacity of CuFe_2_O_4_ was investigated by ultraviolet–visible (UV–Vis) diffuse reflection spectroscopy (DRS). Figure 2E depicts that CuFe_2_O_4_ has a strong photoresponse in the UV region extending to the visible region, making it an ideal catalyst for UV–Vis-mediated PMS activation. Furthermore, this wide photoresponse in the visible region is also important in practical applications where solar light could be utilized as the activation energy for the catalyst.

### 3.3. Performance Evaluation towards BTA Oxidation

**Figure 3:**
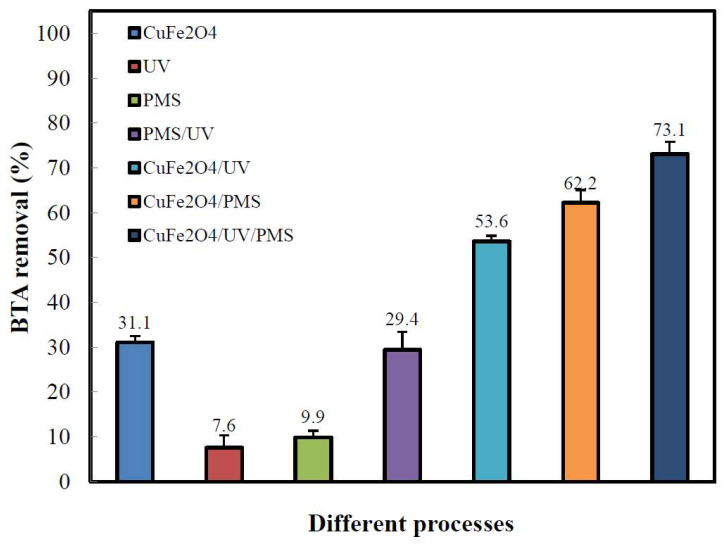
Removal efficiencies of BTA in different reaction systems. Conditions: BTA = 40 mg L^−1^, catalyst (CuFe_2_O_4_) = 0.2 g L^−1^ and PMS = 1.5 mM during the 60 min reaction time.

Figure 3 describes the BTA removal profiles of various catalytic systems under controlled conditions. Notably, in the presence of PMS and UV light separately, only 9.9 and 7.6% of BTA were degraded within 60 min of reaction, respectively, showing a low contribution of direct oxidation in BTA degradation under ambient conditions. Moreover, this is an indication of the stability of BTA towards photolysis, which could be responsible for its persistence in the environment under solar exposure. Additionally, the poor reactivity of the persulfate ions towards BTA oxidation was evident, hence the need for activation. In contrast, the photolysis of PMS improved the oxidation system, with BTA degradation reaching approximately 29.4% after 60 min of UV irradiation. Specifically, UV irradiation triggered the activation of PMS to produce SO_4_^•−^ and ^•^OH radicals through the cleavage of the peroxide bond of PMS. This results in boosted BTA degradation compared to UV photolysis and direct persulfate ion oxidation. Meanwhile, the presence of CuFe_2_O_4_ spinel (0.2 g L^−1^) promoted BTA removal up to 31% in 60 min. This indicates that the redox capacity and adsorption effect of CuFe_2_O_4_ nanoparticles towards BTA molecules provided the necessary catalytic sites for contact with BTA molecules, leading to their degradation [26]. Therefore, the adsorption-desorption equilibrium was established and fixed at 60 min before irradiation for all the tested samples. As expected, BTA degradation was further boosted over CuFe_2_O_4_/UV and CuFe_2_O_4_/PMS coupled systems, reaching 53.6% and 62.2% BTA removal, respectively, demonstrating the catalytic ability of CuFe_2_O_4_ for the activation of PMS and the UV light promoting photocatalytic activity of the material. The synergistic redox action of Cu and Fe sites on the CuFe_2_O_4_ surface induced the PMS decomposition to produce large amounts of SO_4_^•−^ radicals in the CuFe_2_O_4_/PMS oxidation system compared to the individual systems. Similarly, coupling CuFe_2_O_4_ with UV irradiation initiated activation of the photocatalyst to form various reactive species, which collectively contributed to improved photocatalytic degradation of BTA. Despite the improved BTA degradation observed in the various binary systems, the greatest synergistic effect was observed in the binary oxidation system coupled with UV light irradiation. Specifically, the CuFe_2_O_4_/UV/PMS system attained up to 73.1% BTA degradation in 60 min, thus confirming the effectiveness of the photocatalytic PMS oxidation. The enhanced performance of the CuFe_2_O_4_/UV/PMS system can be linked to the collective contribution of photocatalysis and photocatalytic activation of PMS, which yielded powerful SO_4_^•−^ and •OH radicals, responsible for BTA mineralization [33]. Furthermore, it can be inferred that the higher surface area observed in BET analysis coincided with the higher mass transfer of BTA molecules, leading to accelerated reaction rates and mineralization of BTA molecules [9,34]. Moreover, CuFe_2_O_4_ as a highly dispersible catalyst improves the catalyst-BTA interaction and readily provides the photo-generated electrons to active PMS to yield SO_4_^•−^ radicals [17].

To better understand the synergistic effect (SE) between the catalyst (CuFe_2_O_4_), UV, and PMS, the degradation efficiencies of binary and ternary processes were compared to individual processes as outlined in Table 2 [35]. A SE value higher than 1.0 elucidates the existence of a synergy between the components of the oxidation process. Table 2 shows that all the SE values for the binary and ternary processes are greater than 1.0, implying the existence of a synergistic effect in all the studied binary and ternary processes. These results confirm that combining UV, PMS, and CuFe_2_O_4_ provides an accelerated effect on the degradation of BTA in comparison to single and binary systems.

### 3.4. Influence of Operating Parameters on BTA Degradation

#### 3.4.1. CCD Analysis

The CCD experiments and data on the experimental and predicted response values for BTA degradation as a function of catalyst dosage, PMS dosage, BTA initial concentration, and irradiation time are depicted in Table S1. Based on multiple regression analysis, the data showed quadratic polynomial prediction equation agreement in coded factor terms (±α, ±1, 0) as follows:

BTA removal (%) = 58.04 + 8*x*_1_ + 3.01*x*_2_ − 4.38*x*_3_ + 3.92*x*_4_ + 0.51*x*_1_*x*_2_ + 2.23*x*_1_*x*_3_ + 1.83*x*_1_*x*_4_ + 2.76*x*_2_*x*_3_ + 0.42*x*_2_*x*_4_ − 1.34*x*_3_*x*_4_ + 2.37*x*_1_^2^ + 0.95*x*_2_^2^ + 0.09*x*_3_^2^ + 1.61*x*_4_^2^ (7)

Equation (7) shows that the effects of catalyst loading (*x*_1_), PMS dosage (*x*_2_), and irradiation time (*x*_4_) on the predicted response were positive, whereas the effect of initial BTA concentration (*x*_3_) was negative. Additionally, the significant effect of *x*_1_, *x*_2,_ and *x*_4_ on the BTA removal follows the order *x*_1_ *> x*_4_ *> x*_2_. ANOVA analysis (Table S2) shows that the quadratic regression model was highly significant (*p*-value > F-value: F-value = 24.18 and *p*-value = 0.0001) and reliable (Std. Dev. = 3.02). Furthermore, the R^2^ of the quadratic model was 0.9576, providing a 0.957 confidence level for response variability (Figure S1). It was further observed by the normality established between all residuals in the straight line (Figure S2), which can be randomly distributed in the range of ±3.00 (Figure S3), thus indicating an excellent approximation. In addition, R^2^_adj_ of 0.918 was close to R^2^, verifying the goodness-of-fit of the model [36,37,38].

According to the prediction of CCD, the maximum removal of BTA was 81.46% (0.987 desirability), where the optimal values of influencing factors were catalyst loading (0.4 g L^−1^), PMS dosage (2 mM), BTA concentration (20 mg L^−1^), and irradiation time (70 min).

#### 3.4.2. Interaction Analysis of Influential Factors

**Figure 4:**
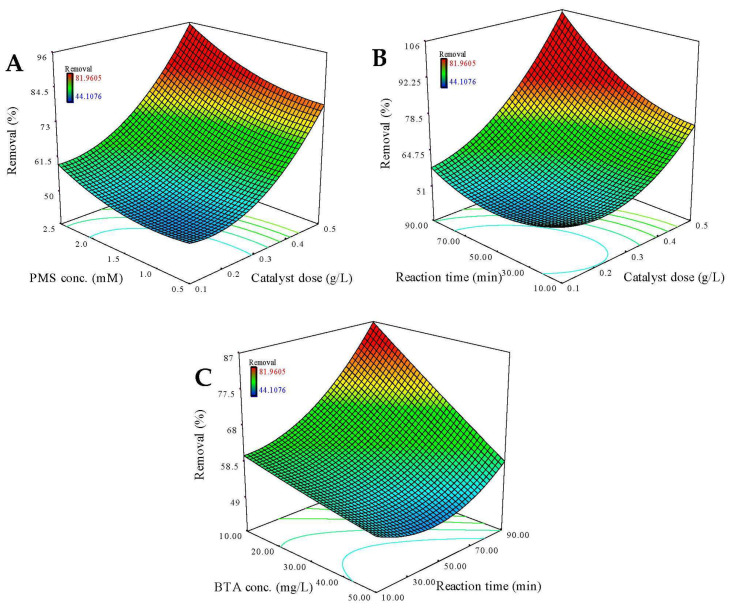
Effect of operating factors on the BTA removal rate over the CuFe_2_O_4_/UV/PMS system: catalyst dosage vs. PMS concentration (**A**), catalyst dosage vs. reaction time (**B**), and BTA concentration vs. reaction time (**C**). Other variables were kept at zero level.

The effects of PMS dosage and catalyst loading on BTA degradation efficiency are shown in Figure 4A. It can be seen that higher initial PMS concentrations and catalyst loads promoted the removal rate of BTA within the specified ranges. This confirms the positive effect of both PMS and catalyst loading on the degradation efficiency. Such increased interaction between the catalyst and PMS results in the generation of a high yield of reactive radicals on the photocatalyst surface. Subsequently, the metal active species, i.e., Cu and Fe sites, decompose PMS to form the radical species, which contribute to the extensive oxidation of 30 mg L^−1^ of BTA within 50 min of irradiation time.

At the constant level of PMS and BTA concentration (Figure 4B), the response surface plot depicts that the catalyst load was not a limiting factor; higher catalyst loading under prolonged irradiation time caused the maximum removal of BTA. The BTA removal efficiency with the CuFe_2_O_4_/UV/PMS process significantly improved from 50.2% to 81.9% upon increasing the dosage of CuFe_2_O_4_ from 0.1 to 0.5 g L^−1^ after 50 min of reaction. This is because more SO_4_^•−^ and ^•^OH radicals were generated via the activation of PMS on the CuFe_2_O_4_ surface (i.e., CuFe_2_O_4 surf_ (e^−^) and CuFe_2_O_4 surf_ (h^+^)) and enhanced the adsorption process of BTA molecules onto the numerous available active sites. These results suggest that the efficacy was affected by the availability of the total surface area as well as the Cu/Fe active site interactions in the catalyst for reaction with PMS during irradiation.

Figure 4C shows the effect of BTA initial concentration and irradiation time on the system's performance. Obviously, the degradation decreased sharply with an increase in BTA concentration from 10 to 50 mg L^−1^. This is largely due to the fact that at constant catalyst loading, the amount of adsorption and catalytic sites for PMS activation and pollutant molecules remains the same despite the increasing amount of BTA molecules. Ultimately, this leads to a decline in the activation rate of PMS and the subsequent degradation of the BTA molecules. Moreover, as the BTA concentration increases, there is an emergence of fierce competition between parent BTA molecules and intermediates for the limited active sites and oxidative radicals on the catalyst surfaces. More specifically, increasing the concentration of BTA on the catalyst surface can decrease photocatalytic reactions that depend upon the direct contact of photo-induced electron/hole pairs to generate active species, which in turn hampers the degradation of BTA. Despite this, an increase in the degradation rate was observed upon extension of the irradiation time. For example, at an initial BTA concentration of 30 mg L^−1^, the degradation rates increased from 50.9% to 75.1% when the reaction time was increased from 10 to 90 min.

### 3.5. Recyclability Performance of the CuFe_2_O_4_/UV/PMS System

**Figure 5:**
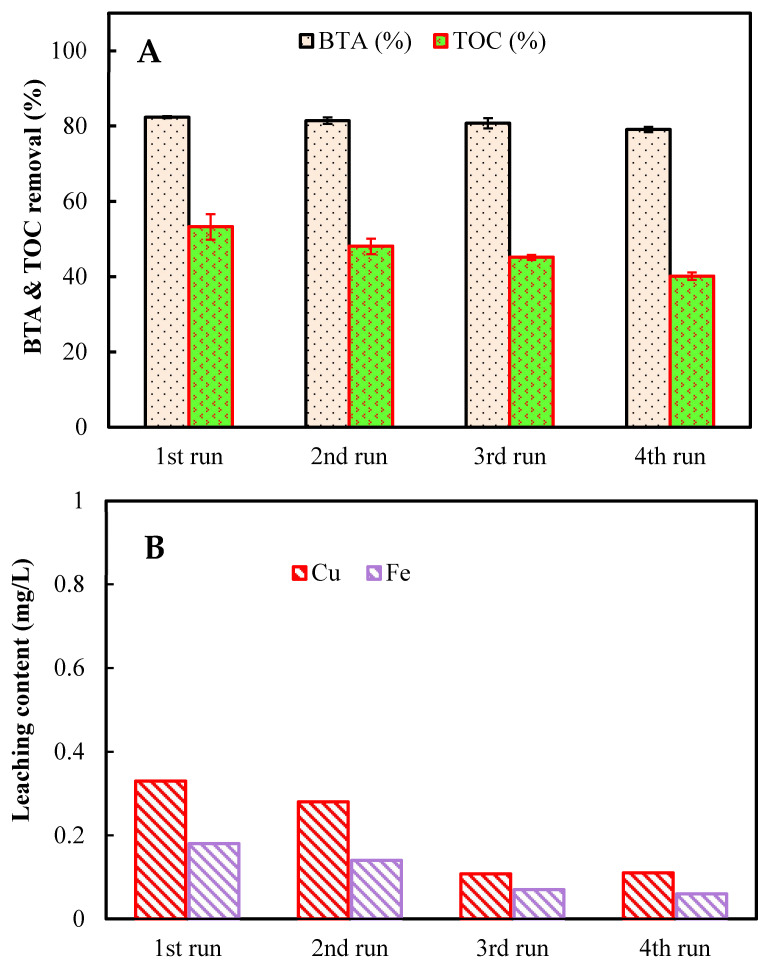
Performance of the recycled catalysts in the CuFe_2_O_4_/UV/PMS system on BTA mineralization (**A**) and Cu and Fe leaching from CuFe_2_O_4_ after each cycle (**B**). Conditions: C_0_ = 20 mg L^−1^, CuFe_2_O_4_ = 0.4 g L^−1^, PMS = 2 mM, and time = 70 min.

Recycling experiments were conducted to determine the stability and multiple reusability potential of the catalyst in the CuFe_2_O_4_/UV/PMS system. This is one of the strengths of the spinel-structured heterogeneous catalytic system. After each cycle, the used catalyst was recovered magnetically, followed by washing with deionized water three times and drying at 80 °C for 1 h. As shown in Figure 5A, the catalytic activity of CuFe_2_O_4_ towards BTA degradation could be maintained at as high as 79% after four photocatalytic cycles (280 min), accompanied by more than 40% TOC removal. Metal leaching detection indicated that CuFe_2_O_4_ also provided less Cu (0.33 mg L^−1^) and Fe (0.18 mg L^−1^) leaching (Figure 5B), corresponding to 0.10% and 0.06% of the total Cu and Fe contents in the catalyst, respectively. These findings reveal that the coordination activities of Cu/Fe pairs in the spinel structure effectively minimized the leaching of metal ions, which led to high stability and catalytic activity over several degradation cycles (less than 4% loss of activity). Despite the simplicity of the process, the CuFe_2_O_4_/UV/PMS system showed a good mineralization rate and removal efficiency of BTA compared to other CuFe_2_O_4_-based catalyst systems previously reported (Table 3).

### 3.6. Feasibility of the Process (Effect of Inorganic Anions)

**Figure 6:**
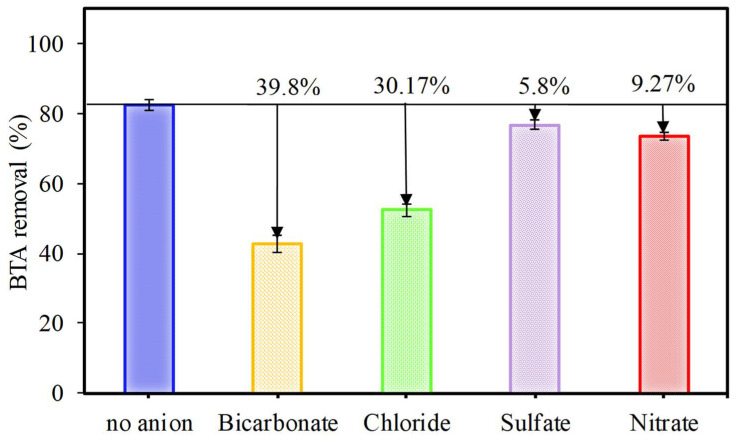
Percentage of BTA removal in the presence of different anions. Conditions: C_0_ = 20 mg L^−1^, CuFe_2_O_4_ = 0.4 g L^−1^, PMS = 2 mM, time = 70 min.

An investigation of the interference of inorganic anions present in natural water and wastewater sources was carried out during BTA degradation to assess the practical applicability of the CuFe_2_O_4_/UV/PMS system. Therefore, BTA degradation was conducted in the presence of 10 mM of major anions, including bicarbonate (HCO_3_^−^), chloride (Cl^−^), sulfate (SO_4_^2−^), and nitrate (NO_3_^−^). Figure 6 reveals that the presence of the inorganic anions caused a suppressive effect on the BTA degradation. Generally, the effect of anions on removal efficiency can be described as follows: (i) catalyzing PMS through electron exchange reactions to generate some radicals with lower redox potential; (ii) competing with pollutant molecules for reactions with free radicals; and (iii) covering the reactive sites and absorbing photons on the CuFe_2_O_4_ surface, leading to the deactivation of reactive sites [32,41]. As shown in Figure 6, the process performance strongly decreased by 42.8% in the presence of HCO_3_^−^ and 52.4% in the presence of Cl^−^. However, SO_4_^2−^ and NO_3_^−^ anions slightly reduced the process efficiency to 76.8% and 73.3%, respectively, which were almost close to the system efficiency without anions. As a result of low reaction kinetics with SO_4_^•−^/^•^OH, the demoting roles of SO_4_^2−^ and NO_3_^−^ as radical scavengers in BTA degradation were lower than those of Cl^−^ and HCO_3_^−^, as shown by Equations (8)–(14). Moreover, through Equations (8) and (9) SO_4_^2−^ can be converted into the aqueous electron (e*^−^*) and highly reactive SO_4_^•−^, respectively. In this way, an aqueous electron (*E°* = −2.9 V) could attack halogenated organic compounds [21].

SO_4_^•−^ + SO_4_^2−^ → S_2_O_8_^2−^ + e^−^ (4.4 × 10^8^ M^−1^ s^−1^) (8)

^•^OH + SO_4_^2−^ → SO_4_^•−^ + OH^−^ (3.5 × 10^5^ M^−1^ s^−1^) (9)

*h*^+^ + SO_4_^2−^ → SO_4_^•−^ (10)

SO_4_^•−^ + e^−^ → SO_4_^2−^ (11)

SO_4_^•−^ + NO_3_^−^ → NO_3_^•−^ + SO_4_^2−^ (5.5 × 10^5^ M^−1^ s^−1^) (12)

^•^OH + NO_3_^−^ → NO_3_^•−^ + OH^−^ (<5 × 10^5^ M^−1^ s^−1^) (13)

*h*^+^ + NO_3_^−^ → NO_3_^•^ (14)

According to Equations (15) and (16), chloride ions can be thermodynamically oxidized by SO_4_^•−^ to chlorine radicals, and therefore chloride ions showed a significant radical scavenging role in the reaction. This decreases the process efficiency from 82.6 to 30.17% (Figure 6). Thus, the lower redox potential of Cl_2_^•−^/2Cl^−^ (*E°* = 2.09 V) and Cl^•^/Cl^−^ (*E°* = 2.47 V) compared to that of SO_4_^•−^/SO_4_^2−^ (*E°* = 2.5–3.1 V) may turn SO_4_^•−^ into slower oxidants (i.e., Cl^•^, ClOH^•−^, and Cl_2_^•−^). PMS (*E°* = 1.75 V) could also react with chloride ions to form sulfate ions in Cl_2_ and HOCl (*E°*_Cl2/2Cl_^−^ = 1.36 V and *E°*_HOCl/Cl_^−^ = 1.48 V) according to Equations (20) and (21) [42].

SO_4_^•−^ + Cl^−^ ↔ SO_4_^2−^ + Cl^•^ (2.3 × 10^8^ M^−1^ s^−1^) (15)

^•^OH + Cl^−^ ↔ ClOH^•−^ (4.2 × 10^9^ M^−1^ s^−1^) (16)

ClOH^•−^ + H^+^ → Cl^•^ + H_2_O (6.1 × 10^9^ M^−1^ s^−1^) (17)

*h*^+^ + Cl^−^ → Cl^•^ (18)

Cl^•^ + Cl^−^ ↔ Cl_2_^•−^ (7.8 × 10^9^ M^−1^ s^−1^) (19)

HSO_5_^−^ + Cl^−^ ↔ SO_4_^2−^ + HOCl (20)

HSO_5_^−^ + 2Cl^−^ + H^+^ ↔ SO_4_^2−^ + Cl_2_ + H_2_O (21)

As shown in Figure 6, a significant decrease (39.8%) in the degradation efficiency was observed in the presence of HCO_3_^−^. This may be attributed to the fast scavenging of the photo-oxidizing species (SO_4_^•−^, ^•^OH, and h^+^) by HCO_3_^−^, as well as the production of CO_3_^•−^ via Equations (22)–(24), which mitigate the consumption of BTA [18]. Despite the high availability of HCO_3_^•−^/CO_3_^•−^, there was no enhancement effect on BTA removal because of their low oxidation capability [43]. Additionally, HCO_3_^−^ was found to inhibit PMS activation.

SO_4_^•−^ + HCO_3_^−^ → SO_4_^2−^ + CO_3_^•−^ + H^+^ (1.6 × 10^6^ M^−1^ s^−1^) (22)

^•^OH + HCO_3_^−^ → CO_3_^•−^ + H_2_O (8.5 × 10^6^ M^−1^ s^−1^) (23)

*h*^+^ + HCO_3_^−^ → HCO_3_^•−^ (24)

### 3.7. Radical Scavenging Experiments

**Figure 7:**
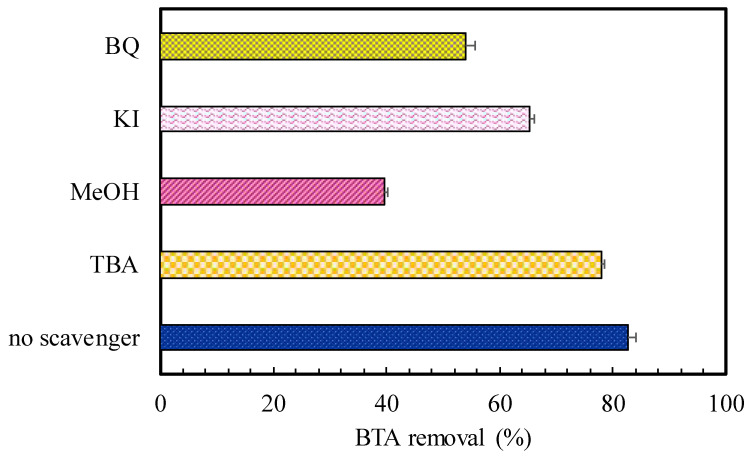
The effects of different radical scavengers on BTA degradation. Conditions: C_0_ = 20 mg L^−1^, CuFe_2_O_4_ = 0.4 g L^−1^, PMS = 2 mM, time = 70 min.

Quenching tests were conducted to identify the predominant active species in the CuFe_2_O_4_/UV/PMS system responsible for BTA degradation (Figure 3). The quenching agents, including TBA, MeOH, BQ, and KI, were used in concentrations of 10 mM to trap ^•^OH, SO_4_^•−^, O_2_^•−^, and *h*^+^, respectively [9,16]. According to the different quenching reaction rates, MeOH can similarly quench both SO_4_^•−^ and ^•^OH (*k*_MeOH/•OH_ = 9.7 × 10^8^ M^−1^ s^−1^, *k*_MeOH/SO4•−_ = 3.2 × 10^6^ M^−1^ s^−1^), while TBA, with 1000 times higher reactivity towards ^•^OH compared to SO_4_^•−^ (*k*_TBA/•OH_ = 3.8–7.6 × 10^8^ M^−1^ s^−1^, *k*_TBA/SO4•−_ = 4.0–9.1 × 10^5^ M^−1^ s^−1^), preferentially scavenge ^•^OH radicals [44]. Figure 7 displays that the degradation efficiency decreased from 82.6% (without any quenchers) to 78 and 39.6% in the system with TBA and MeOH, respectively. Furthermore, it was found that the addition of KI resulted in a 65.4% reduction in process efficiency, while BQ led to a much stronger inhibitory effect (a 54.0% decrease), even more than TBA. These results confirmed the production of the reactive species ^•^OH, SO_4_^•−^, O_2_^•−^, and *h*^+^, and among them, SO_4_^•−^ played the most predominant role during the BTA decomposition process in the CuFe_2_O_4_/UV/PMS system.

### 3.8. Mechanistic Discussion

**Scheme 1:**
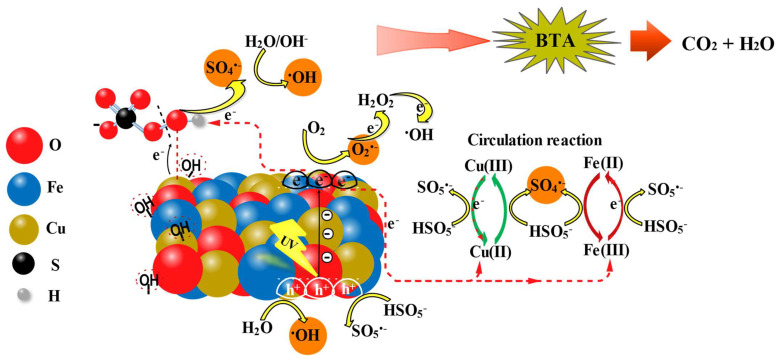
Schematic diagram of the reaction mechanism for degradation of BTA by the CuF_2_O_4_/UV/PMS process.

Based on the active species capture experiments and some previous studies [9,13,22], a possible mechanism (Scheme 1) has been proposed to explain the charge transfer routes during the photocatalytic PMS activation leading to BTA degradation. The absorbed UV light caused the excitation of electrons in the valence band (VB) of the catalyst to the conduction band (CB) to produce photo-induced electron and hole pairs (e^−^/h^+^) as depicted in Equation (25). Subsequently, the conduction band electrons (e_CB_^−^) of CuFe_2_O_4_ could quickly reduce dissolved O_2_ to O_2_^•−^ radicals (Equation (26)) based on the standard redox potential difference (*E*^0^_e_CB__^−^ = −0.48 eV vs. NHE compared to *E*^0^_O_2_/O_2__^•−^ = −0.46 eV vs. NHE) [22]. At the same time, h^+^ could react with adsorbed H_2_O or OH^−^ to form ^•^OH (Equation (27)) [45]. O_2_^•−^ could also trigger a series of redox reactions to produce ^•^OH (Equations (28) and (29)) [9].

CuFe_2_O_4_ + h*v* → h_VB_^+^ + e_CB_^−^ (25)

CuFe_2_O_4_ (e_CB_^−^) + O_2_ → O_2_^•−^ (26)

CuFe_2_O_4_ (h_VB_^+^) + H_2_O → ^•^OH + H^+^ (27)

O_2_^•−^ + e_CB_^−^ + 2H^+^ → H_2_O_2_ (28)

H_2_O_2_ + e_CB_^−^ → ^•^OH + OH^−^ (29)

Additionally, it was observed that PMS further enhanced the catalytic reaction, which could be attributed to the chemical stability arising from the redox cycles of surface active centers. It was concluded that the process of CuFe_2_O_4_ catalyzed PMS for generating reactive species was initially related to the binding of hydroxyl groups (−OH) obtained from the dissociation of water on the surface metal (Fe or Cu) sites in the CuFe_2_O_4_ catalyst [27]. In this regard, HSO_5_^−^ could form a bond with the surface Cu(II) of the catalyst via surface −OH displacement and generate a Cu(II)-(OH)OSO_3_^−^ intermediate by the inner-sphere complexation (Equation 30). Therefore, the favorability of the electron transfer from Cu(II), with the obtained high electron density, to OH of HSO_5_^−^ could lead directly to the production of SO_4_^•−^ radical and a new surface −OH group that could bond to a higher valence Cu(III) ion (Equation (31)). Supposing that ≡Cu(III)−OH oxidized HSO_5_^−^ to SO_5_^•−^ bonded to copper initial valence (i.e., Cu(II)) (Equation (32)), then the combination of surface SO_5_^•−^ moieties would again generate SO_4_^•−^ via Equation (33). The efficient participation in the redox process could result in the reductant character for Fe(II), reducing ≡Cu(III) to ≡Cu(II) (Equation (34)), since *E*^0^_≡Cu(III)/≡Cu(II)_ = 2.3 V is much higher than *E*^0^_≡Fe(III)/≡Fe(II)_ = 0.77 V. The resulting ≡Fe(III) would be possibly turned to ≡Fe(II) during the process of HSO_5_^−^ reduction (Equation 35), which would be subsequently oxidized to ≡Fe(III) by the production of SO_4_^•−^ radicals (Equation (36)). Thus, redox mediators, i.e., Cu(II-III-II) and Fe(III-II-III), by maintaining the reaction cycles not only enhance the activity of CuFe_2_O_4_ via Equation (34) but also contribute to the further decomposition of PMS through Equations (35) and (36). From the hydroxylation of SO_4_^•−^,^•^OH can be released according to Equations (37) and (38) [46]. Photocatalytic activation of PMS, under the action of photoinduced electrons, opened another route for the direct generation of ^•^OH and SO_4_^•−^ radicals (Equation (39)). In this way, the photoinduced holes could be captured by PMS to produce SO_4_^•−^ via subsequent self-reaction of SO_5_^•−^ radicals (Equations (40) and (41)). On the other hand, during the reaction, photo-induced electrons under the different valence states of Cu–Fe could create a new equilibrium to obtain the cyclic Cu(III)/Cu(II) and Fe(III)/Fe(II) (Equation (42)). This process can lead to fast photocharge transfer in the presence of PMS and may explain the higher reactivity compared to the binary systems, namely CuFe_2_O_4_/UV and CuFe_2_O_4_/PMS (Figure 3). Thus, when combined with photocatalysis, PMS oxidation showed a synergistic effect by producing more free active species. A negligible amount of SO_4_^•−^, ^•^OH, O_2_^•−^, and h^+^ radicals that could have leached from the surface-bound layer to the solution bulk in the CuFe_2_O_4_/UV/PMS system has little effect on the mineralization of BTA (Equation (43)).

≡Cu(II)˗˗˗OH^−^ + HSO_5_^−^ → ≡Cu(II)˗˗˗(OH)˗OSO_3_^−^ + OH^−^ (30)

≡Cu(II)˗˗˗(OH)˗OSO_3_^−^ → ≡Cu(III)˗˗˗OH^−^ + SO_4_^•−^ (31)

≡Cu(III)˗˗˗OH^−^ + HSO_5_^−^ → ≡Cu(II)˗˗˗SO_5_^•−^ + H_2_O (32)

2≡Cu(II)˗˗˗SO_5_^•−^ + 2H_2_O → 2≡Cu(II)˗˗˗OH^−^ + O_2_ + 2SO_4_^•−^ + 2H^+^ (33)

≡Cu(III)˗˗˗OH^−^ + ≡Fe(II) → ≡Cu(II)˗˗˗OH^−^ + ≡Fe(III) (34)

≡Fe(III) + HSO_5_^−^ → ≡Fe(II) + SO_5_^•−^ + H^+^ (35)

≡Fe(II) + HSO_5_^−^ → ≡Fe(III) + SO_4_^•−^ + OH^−^ (36)

SO_4_^•−^ + H_2_O → ^•^OH + SO_4_^2−^ + H^+^ (37)

SO_4_^•−^ + OH^−^ → ^•^OH + SO_4_^2−^ (38)

e_CB_^−^ + HSO_5_^−^ → SO_4_^•−^ + OH^−^ (39)

h_VB_^+^ + HSO_5_^−^ → SO_5_^•−^ + H^+^ (40)

2SO_5_^•−^ → 2SO_4_^•−^ + O_2_ (41)

Cu(III)/Fe(III) + e_CB_^−^ → Cu(II)/Fe(II) (42)

SO_4_^•−^/^•^OH/O_2_^•−^/h^+^ + BTA → products → CO_2_ + H_2_O + NH_3_ (43)

### 3.9. Reaction Pathway of BTA Degradation

**Figure 8:**
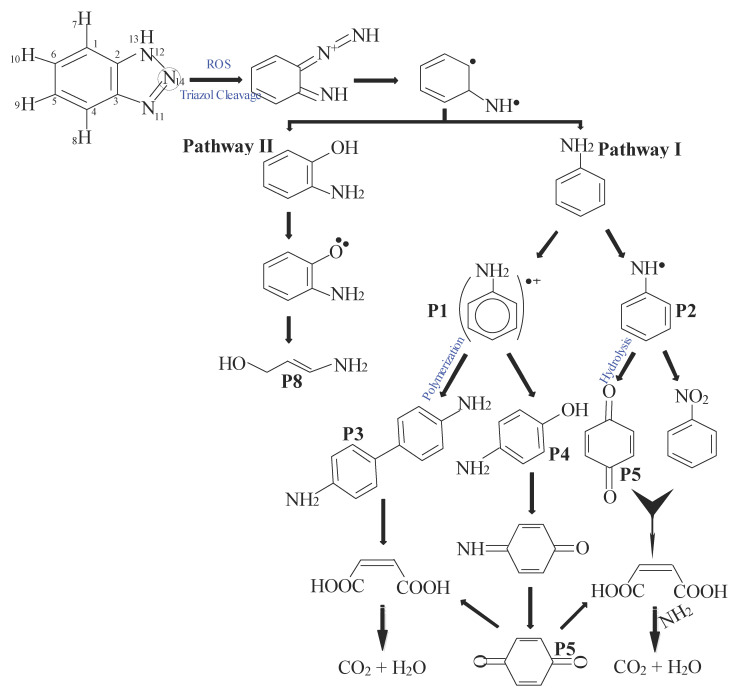
Schematic degradation pathways of BTA during the CuFe_2_O_4_/UV/PMS process based on the potential consequences.

The BTA degradation pathway in the CuFe_2_O_4_/UV/PMS system was proposed and described in Figure 8 based on the literature [47,48,49]. As shown in Figure 8, radicals attack the active sites of the BTA molecule and may first cleave the triazole ring of the molecule at site N14. In this way, the nitrogen double bond was destroyed, and following the N–NH bond dissociation, it would form the yellow intermediate diazoimine. This primary diazo intermediate was a transient product, resulting in its stepwise oxidation to release a nitrogen molecule and produce the colorless biradical intermediate under irradiation. Based on the recombination reaction, aniline could be formed [5,47], which is in accordance with the identification of HPLC analysis using standard aniline. The structure and chemical formula of the identified intermediates are given in Table S3. In pathway (I), the intermediates aniline radicals (P1) and benzoquinonimine (P2) appeared. In this step, the aniline radicals could undergo direct polymerization to form dianiline (P3) leading to the opening of the aromatic ring of aniline to form maleic acid (P7) [48]. Alternatively, the aniline radicals could produce intermediate 4-aminophenol (P4), which is the main precursor for the formation of benzoquinone (P5) via the action of ROS. Additionally, benzoquinone (P5) can be formed through the hydrolysis reaction of benzoquinonimine (P2) that occurs concurrently with deamination. Moreover, nitrobenzene (P6) was observed through the catalytic activity of ROS on benzoquinonimine (P2), leading to the formation of maleic acid (P7) by further deamination, especially losing NO_3_^−^ and finally being converted into CO_2_ and H_2_O [49]. Under the attack of the ROS, the deprotonation process on the external carbons could also occur to produce the ring-cleavage intermediate 3-aminoprop-2-en-1-ol (P8) (pathway II) [2].

## 4. Conclusions

Herein, photosynergistic activation of PMS with a heterogenous CuFe_2_O_4_ catalyst was assessed to study BTA degradation under UV light. The novel-designed system exhibited a 1.17- and 1.3-fold increase in catalytic activity for BTA degradation, reaching a high efficiency of 73%. This result was higher than that of CuFe_2_O_4_/PMS (62.2%) and CuFe_2_O_4_/UV (53.6%) binary systems, respectively, indicating a synergy effect between UV, PMS, and the catalyst. The radical quenching tests conducted in this study demonstrated the generation of multiple radicals, including ^•^OH, SO_4_^•−^, O_2_^•−^, and *h*^+^. These radicals were generated through the conversion of Cu(II)/Cu(III) and Fe(III)/Fe(II) on the surface of CuFe_2_O_4_ with SO_4_^•−^ playing a dominant role in the radical generation process. This resulted in negligible leaching of metals and the excellent recyclability of synthesized catalysts. The samples prepared in this study demonstrated excellent catalytic performance and low levels of Fe and Cu leaching over the course of four consecutive runs. Collectively, this study presented a feasible approach for improving the catalytic performance of wastewater treatment systems for environmental remediation.

## Figures and Tables

**Table 1:** Four independent factors and their ranges at various levels.

Independent Factors	Unit	Symbols	Ranges and Levels				
			−α	Low (−1)	Middle (0)	High (+1)	+α
Catalyst loading	g L^−1^	*x*_1_	0.1	0.2	0.3	0.4	0.5
PMS dosage	mM	*x*_2_	0.5	1	1.5	2	2.5
Initial BTA concentration	mg L^−1^	*x*_3_	10	20	30	40	50
Irradiation time	min	*x*_4_	10	30	50	70	90

**Table 2:** Summary of the synergistic effect of different systems towards BTA elimination.

No.	SE Equation	SE Value
1	$SE=\frac{k_{PMS/UV}}{k_{PMS}+k_{UV}}$	1.68
2	$SE=\frac{k_{Cu{Fe}_{2}O_{4}/UV}}{k_{Cu{Fe}_{2}O_{4}}+k_{UV}}$	1.38
3	$SE=\frac{k_{Cu{Fe}_{2}O_{4}/PMS}}{k_{Cu{Fe}_{2}O_{4}}+k_{PMS}}$	1.51
4	$SE=\frac{k_{Cu{Fe}_{2}O_{4}/UV/PMS}}{k_{Cu{Fe}_{2}O_{4}}+k_{PMS}+k_{UV}}$	1.50
5	$SE=\frac{k_{Cu{Fe}_{2}O_{4}/UV/PMS}}{k_{Cu{Fe}_{2}O_{4}/UV}+k_{PMS}}$	1.15
6	$SE=\frac{k_{Cu{Fe}_{2}O_{4}/UV/PMS}}{k_{CuF{Fe}_{2}O_{4}/PMS}+k_{UV}}$	1.04

**Table 3:** The degradation of pollutants in other similar oxidation systems with various catalysts.

System	Pollutant (mg/L)	Catalyst (g/L)	Oxidant (mM)	Removal Efficiency (%)	Mineralization Rate (%)	Metal Ion Leaching (mg/L)	Ref.
CuFe@NG/PMS	Sulfamethoxazole (10)	0.3	0.4	93.15 in 60 min	31.96	[Cu] = 0.25, [Fe] = -	[39]
CuFe_2_O_4_@GO/PMS	Methylene blue (20)	0.2	0.8	93.3 in 30 min	-	[Cu] = 0.3, [Fe] = 0.3	[32]
OVs-CFEp/Vis/PMS	Sulfamethazine (10)	1	0.3	95 in 90 min	56	[Cu] < 0.1, [Fe] < 0.1	[9]
CuFe_2_O_4_ 3DPs/Vis/PMS	Tetracycline hydrochloride (20)	0.25	0.2	93 in 30 min		[Cu] = 0.2, [Fe] = 0.2	[22]
CuFe_2_O_4_-CoFe_2_O_4_/PMS	BPA (20)	0.2	1	98.7 in 110 min	72.5		[21]
CuFe_2_O_4_/kaolinite/PMS	BPA (50)	0.5	0.5	97 in 60 min	55	[Cu] = 0.27, [Fe] = 0.01	[17]
CuFe_2_O_4_/Biochar/Ag_3_PO_4_/Vis	Phenol (20)	0.15	-	100 in 18 min			[40]
CN/BVO/CFO/Vis/PMS	Levofloxacin (10)	0.2	1	96.2 in 60 min	67	[Cu] = 0.08, [Fe] = 0.02	[13]
CuFe_2_O_4_/MnO_2_/Vis/PMS	Phenol (100)	0.5	0.5	100 in 30 min	62.2	[Cu] = 0.026, [Fe] = 0.012	[37]
CuFe_2_O_4_/UV/PMS	BTA (20)	0.4	2	82.3 in 70 min	53.2	[Cu] = 0.33, [Fe] 0.18	This study

## Data Availability

Data will be made available on request.

## Supplementary Materials

The following supporting information can be downloaded at: https://www.mdpi.com/article/10.3390/toxics11050429/s1. Table S1: CCD experiments and observed and predicted removal efficiencies using CuFe_2_O_4_/UV/PMS system; Table S2: Results of ANOVA for response surface quadratic model; Figure S1: Correlation between predicted and experimental values of BTA degradation variability; Figure S2: The normal probability plot of the internally studentized residuals; Figure S3: The experimental run number versus studentized residual data; Table S3 The structure and chemical formula of detected intermediates from BTA degradation in the CuFe_2_O_4_/UV/PMS system.
