# Supplementary material for: Synergetic Photocatalytic Peroxymonosulfate Oxidation of Benzotriazole by Copper Ferrite Spinel: Factors and Mechanism Analysis
Source: Toxics. 2023 May 4;11(5):429. doi: 10.3390/toxics11050429 (PMC10222566; doi:10.3390/toxics11050429)
Supplement: Supplementary file 1 [file toxics-11-00429-s001.zip › toxics-2343418-supplementary.pdf]

Supporting materials

Table S1. CCD experiments and observed and predicted removal efficiencies using $CuFe_2O_4$/UV/PMS system.

Run	Independent variables				Actual (Re%)	Predicted (Re%)	Residual
	x_1	x_2	x_3	x_4			
1	0.2	2	20	30	55.97	57.56	-1.59
2	0.3	1.5	30	50	57.31	58.04	-0.73
3	0.2	2	40	30	55.24	52.56	2.68
4	0.2	1	40	70	44.11	43.54	0.56
5	0.3	1.5	30	50	58.06	58.04	0.022
6	0.4	2	40	30	72.39	70.38	2.01
7	0.3	0.5	30	50	52.35	55.83	-3.48
8	0.4	1	40	30	59.42	58.63	0.79
9	0.2	1	40	30	46.42	42.87	3.54
10	0.3	1.5	10	50	67.67	67.15	0.52
11	0.5	1.5	30	50	81.96	83.50	-1.54
12	0.2	2	40	70	52.74	54.89	-2.15
13	0.2	1	20	70	63.27	64.94	-1.68
14	0.4	1	40	70	68.54	66.62	1.93
15	0.3	1.5	30	10	50.99	56.67	-5.68
16	0.1	1.5	30	50	50.22	51.51	-1.29
17	0.3	2.5	30	50	68.51	67.87	0.64
18	0.3	1.5	50	50	46.30	49.65	-3.35
19	0.2	2	20	70	66.95	65.24	1.7
20	0.4	2	20	30	68.41	66.48	1.93
21	0.3	1.5	30	50	57.66	58.04	-0.38
22	0.3	1.5	30	90	75.17	72.33	2.84
23	0.4	2	20	70	78.27	81.47	-3.21
24	0.4	1	20	30	68.27	65.78	2.48
25	0.4	1	20	70	78.93	79.11	-0.18
26	0.3	1.5	30	50	58.65	58.04	0.61
27	0.3	1.5	30	50	58.25	58.04	0.21
28	0.3	1.5	30	50	58.31	58.04	0.27
29	0.4	2	40	70	80.18	80.02	0.16
30	0.2	1	20	30	61.26	58.92	2.34

Table S2. Results of ANOVA for response surface quadratic model.

Source	Sum of squares	df	Mean Square	F Value	p-value Prob > F
Model	3081.86	14	220.13	24.18	< 0.0001
x_1 – Catalyst dosage	1535.24	1	1535.24	168.65	< 0.0001
x_2 – PMS Conc.	217.56	1	217.56	23.9	0.0002
x_3 – BTA Conc.	459.54	1	459.54	50.48	<0.0001
x_4 – Reaction time	368.06	1	368.06	40.43	<0.0001
$x_1 x_2$	4.24	1	4.24	0.47	0.5053
$x_1 x_3$	79.22	1	79.22	8.7	0.0099
$x_1 x_4$	53.47	1	53.47	5.87	0.0285
$x_2 x_3$	122.03	1	122.03	13.41	0.0023
$x_2 x_4$	2.76	1	2.76	0.3	0.59
$x_3 x_4$	28.6	1	28.6	3.14	0.0966
x_1^2	153.6	1	153.6	16.87	0.0009
x_2^2	24.88	1	24.88	2.73	0.1191
x_3^2	0.22	1	0.22	0.024	0.8778
x_4^2	71.48	1	71.48	7.85	0.0134
Residual	136.54	15	9.1		
Lack of Fit	135.37	10	13.54	57.94	0.0002
Pure Error	1.17	5	0.23		
Correction Total	3218.41	29			
Std. Dev.	3.02		R-squared	0.9576	
Mean	62.06		Adj. R-squared	0.918	
C.V.	4.86		Pred. R-squared	0.7572	
Press	781.44		Adeq. Precision	19.045	

R^2 = 0.9576; R^2_{adj} = 0.918; R^2_{pred} = 0.7572; adequacy precision = 19.045; C.V.% = 4.86.

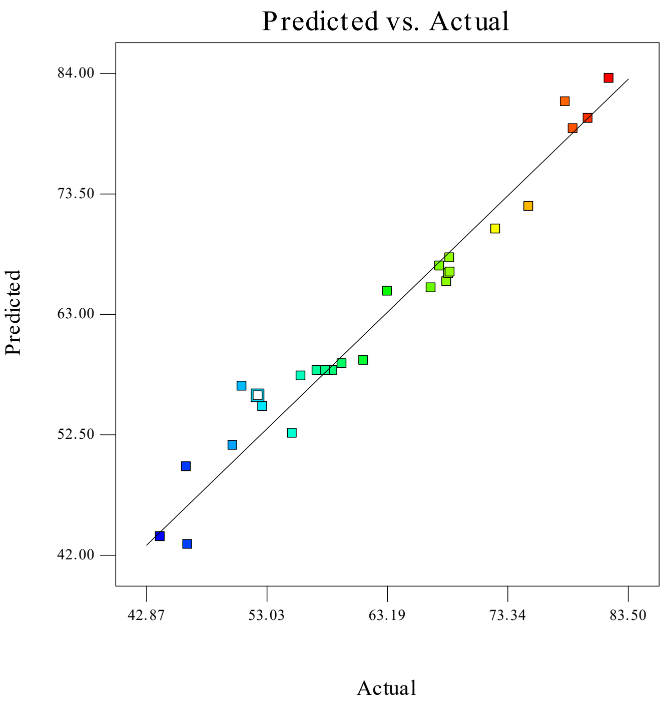

Figure S1. Correlation between predicted and experimental values of BTA degradation variability.

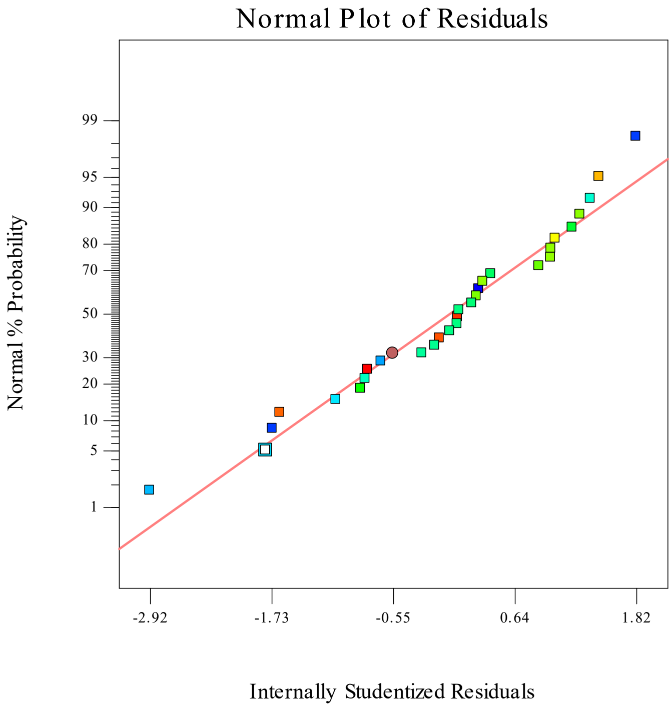

Figure S2. The normal probability plot of the internally studentized residuals.

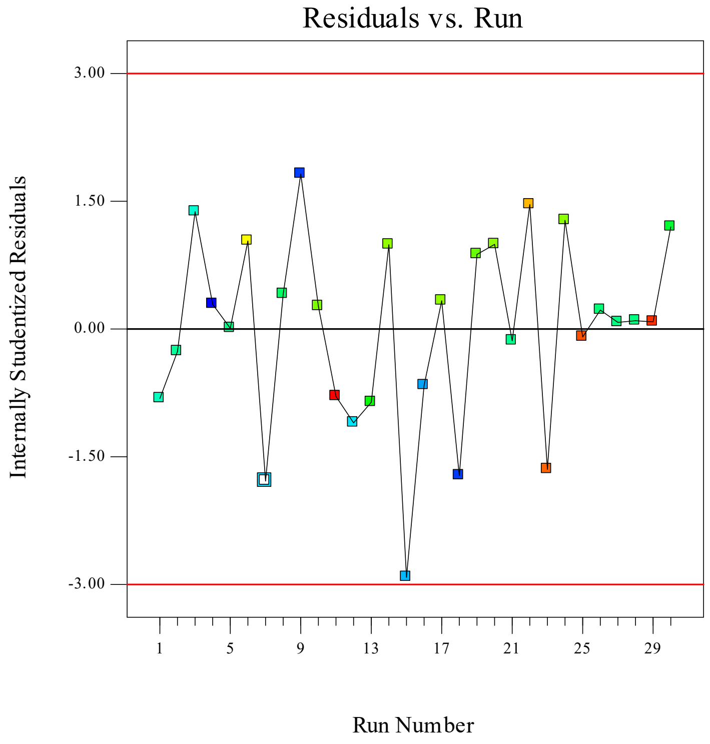

Figure S3. The experimental run number versus studentized residual data.

Table S3. The structure and chemical formula of detected intermediates from BTA degradation in the $CuFe_2O_4$/UV/PMS system.

Compound	(m/z)	Structural formula	Molecular formula
aniline	93	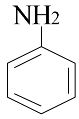	C6H7N
benzoquinonimine	92	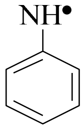	C6H6N
dianiline	184	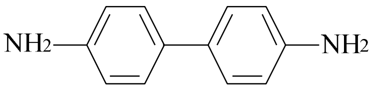	C12H14N
4-aminophenol	109	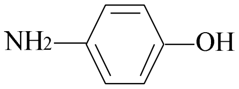	C6H7NO
nitrobenzene	123	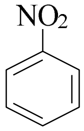	C6H5NO2
benzoquinone	108	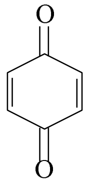	C6H4O2
3-aminoprop-2-en-1-ol	73	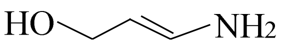	C3H7NO
maleic acid	116	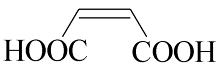	C4H4O4
